# A Comparative Performance Evaluation of YOLO-Type Detectors on a New Open Fire and Smoke Dataset

**DOI:** 10.3390/s24175597

**Published:** 2024-08-29

**Authors:** Constantin Catargiu, Nicolae Cleju, Iulian B. Ciocoiu

**Affiliations:** Faculty of Electronics, Telecommunications and Information Technology, Gheorghe Asachi Technical University of Iasi, Bd. Carol I 11A, 700506 Iasi, Romania; constantin.catargiu@yahoo.com (C.C.); nikcleju@etti.tuiasi.ro (N.C.)

**Keywords:** fire and smoke detection, mAP, YOLO object detectors, real-time operation

## Abstract

The paper introduces a new FireAndSmoke open dataset comprising over 22,000 images and 93,000 distinct instances compiled from 1200 YouTube videos and public Internet resources. The scenes include separate and combined fire and smoke scenarios and a curated set of difficult cases representing real-life circumstances when specific image patches may be erroneously detected as fire/smoke presence. The dataset has been constructed using both static pictures and video sequences, covering day/night, indoor/outdoor, urban/industrial/forest, low/high resolution, and single/multiple instance cases. A rigorous selection, preprocessing, and labeling procedure has been applied, adhering to the findability, accessibility, interoperability, and reusability specifications described in the literature. The performances of the YOLO-type family of object detectors have been compared in terms of class-wise Precision, Recall, Mean Average Precision (mAP), and speed. Experimental results indicate the recently introduced YOLO10 model as the top performer, with 89% accuracy and a mAP@50 larger than 91%.

## 1. Introduction

Fire is one of the most hazardous and potentially fatal calamities, threatening the environment, properties, and human lives [1]. According to the most recent report of the Center for Fire Statistics affiliated with the International Association of Fire and Rescue Services, 3.7 million fire-related calls were registered in 2022 in 55 countries surveyed. About 19,600 people died and 55,600 were injured during these incidents, including a significant number of on-duty firefighters [2].

The adverse effects on the environment and the global community of both forest/wildfires and those occurring within urban areas are significant and cannot be overstated. Given the escalating pace of population concentration and an increased emphasis on safety, early fire detection has become a primary concern in developing smart cities. Classical sensor-based detection systems [3] may not be suitable in certain scenarios, such as vast coverage areas or untamed locations, as they can generate numerous false alerts and sometimes fail to trigger. While effective for identifying small flames, they often prove ineffective for larger, rapidly spreading fires. On the other hand, smoke detectors possess a limited ability to differentiate between different types of smoke, such as those generated by pollution, cigarettes, cooking fumes, and actual fire [4]. For example, according to the Duisburg Fire Department, only 84% of fire detectors’ automatically triggered fire alarm calls correspond to real fire situations [5]. Additionally, 44% of false positive alarms are triggered by aerosols, with nearly 20% being dust and water aerosols [6].

A viable alternative to traditional fire alarm systems is offered by modern AI/computer vision solutions [7]. More specifically, deep learning fire/smoke detection models can automatically learn relevant features, effectively overcoming the redundancy and interference associated with hand-crafted engineering. They enable the real-time identification and precise localization of fire-related incidents, providing clear performance metrics regarding confidence scores and speed while minimizing false alarm rates. 

The present paper introduces a new open fire/smoke dataset with more than 22,000 images and 93,000 distinct instances compiled from static pictures and video sequences. The dataset was generated based on a rigorous selection, preprocessing, and labeling procedure that complies with the FAIR (findability, accessibility, interoperability, and reusability) specifications described in [8]. Providing data in multiple formats, the use of a multiple-step annotation procedure for images covering a broad range of realistic situations, along with a curated set of challenging cases exhibiting real-life circumstances when specific image patches may be erroneously detected as fire/smoke presence represent the main characteristics of this challenging dataset. Data are available on the GitHub platform [9], along with the Python code for downloading and a statistical evaluation of the distribution of the various image categories. 

We comparatively evaluated the performances of several recent members of the YOLO (You Only Look Once) family of efficient real-time object detectors [10] on the proposed dataset. YOLOv5–v10 and YOLO-NAS models were compared in terms of class-wise Precision, Recall, Mean Average Precision (mAP), and speed. The performances of the models were evaluated through extensive experimentation on realistic case studies, showcasing the practical deployment of the system in real-world settings. The various sources of variability include indoor/outdoor environments, urban/industrial/ship/forest, low/high resolution, and single/multiple instance scenarios. The GitHub project page also includes Python scripts that generate metrics containing valuable information for the inference tasks. Those metrics provide useful info such as the total number of detections for each class, the average confidence of these detections per class, and the number of incorrect predictions made by each model. Additionally, we computed the average speed (fps), the total inference time, and the total number of detections across all classes.

The main contributions of the paper are as follows:

- The generation of a novel large open dataset that includes challenging images that may (but should not) be detected as fire instances;
- A two-step annotation procedure that combines an initial automatic solution based on the use of the YOLO8 detection model with a second manual correction and bounding box adjustment phase that also removes labeling errors;
- A performance comparison of seven YOLO models, including the most recent YOLO10 member of this family of object detectors;
- A comparative analysis of the detection efficiency of the analyzed models for daylight vs. night-time images.

The following sections describe existing datasets and machine learning models presented in the literature for accurately identifying fire and/or smoke, along with the protocol used to generate the new FireAndSmoke dataset. The experimental results reveal comparative performances of seven distinct members of the YOLO family of object detector models, while conclusions and suggestions for further work are finally outlined. 

## 2. Datasets and Models for Fire/Smoke Identification

### 2.1. Fire and Smoke Datasets

Existing public datasets exhibiting fire and/or smoke scenes vary to a large extent in terms of the number of images/instances, content (fire and/or smoke, non-fire/smoke), resolution, and weather conditions. Additional sources of variability include the type of sensors used (visible spectrum/infrared cameras), the actual acquisition setup (space-borne/air-borne/ground-borne cameras), or the specific environment scenario (wildfire/forest/urban/industrial/ship). Table 1 illustrates a list of public datasets largely used in the literature. 

Some papers have considered fire or smoke scenarios only, others aim at discriminating fire/smoke scenes from general non-fire/smoke situations, while a relatively short list of datasets includes images affected by poor weather conditions (haze, fog, or rain). Moreover, specific environments are only considered in many cases, such as forest fires or flames bursting on ships.

The present FireAndSmoke dataset contains a broad range of fire and smoke scenarios, such as indoor fires, building fires, car fires, forest/wildfires, and ship fires. The actual images largely vary in terms of resolution, lighting, and weather conditions. The new dataset additionally contains challenging situations when image patches could mistakenly be classified as fire instances. Those are marked as the “other” category and include natural and artificial light sources such as sunsets/sunrises, different types of clouds, streetlamps, bonfire glows, reflections, vehicle headlights, emergency lights of various vehicles, or lights from public electronic screens. 

### 2.2. Machine Learning Models for Fire/Smoke Identification

Fire/smoke identification strategies typically follow one of three approaches, namely classification, detection, or segmentation, while combinations of those have also been considered. Classification methods process the images as a whole, aiming to separate the ones containing fire and/or smoke instances. Detection-based solutions take a step further by additionally indicating the actual localization and extension of the regions of interest within an image. At the same time, segmentation accurately delineates the exact set of pixels that belong to fire or smoke patches. From a practical perspective, detection- and segmentation-oriented approaches are preferred since they may offer invaluable information regarding the spread, dynamics, and possible fast expansion toward critical areas (houses, fuel storage facilities, etc.).

Classical machine learning algorithms targeting fire/smoke identification tasks typically rely on feature extraction techniques such as flame-specific chromatograms, shapes, textures, and flame motion. For instance, in [18], the authors selected the YCbCr color space to differentiate the luminance from the chrominance information better, yielding superior results compared to RGB-based solutions. Poobalan et al. [19] employed RGB and HSL filters along with a bag-of-features (BoF) model to detect fire color, improving classification performances. The use of the Lucas–Kanade optical flow algorithm was suggested in [20], with potential smoke areas determined using a background estimate and a color-based decision procedure. 

In the realm of flame-texture feature extraction, Cui et al. [21] employed wavelet analysis and gray-level co-occurrence matrices (GLCMs) to analyze fire smoke texture. Yu et al. [22] proposed a real-time fire smoke detection approach based on gray-level co-occurrence matrices (GLCMs), incorporating a neural network for texture classification. Chino et al. [23] innovated by combining color features and texture classification in superpixel areas for static image fire detection. Ye et al. [24] introduced a dynamic texture descriptor using surface transform and a hidden Markov tree (HTM) model.

Through their innovative approach, Toulouse et al. [25] identified various geometrical features of flames, including location, rate of spread, length, and surface area. They categorized pixels representing fire based on color, while non-refractive pixels were utilized to detect smoke, sorted by their average intensity. Avgeris et al. [26] developed an edge computing framework for early fire detection, streamlining border identification significantly. However, these computer-vision-based frameworks were primarily employed on static fire images. Recently, researchers have employed fast Fourier transform (FFT) and wave variation techniques to analyze wildfire boundaries in videos [27], albeit with effectiveness limited to specific scenarios.

Researchers in [28] developed a method for fire detection by analyzing smoke and flame motion with linear dynamic systems. By incorporating color, motion, and spatial-temporal features into their model, they achieved high detection rates and a significant reductions in false alarms. Researchers also analyzed forest fire locations by examining the spatial and temporal dynamics of fire textures [29]. Hybrid surface descriptors were employed in a static texture analysis to generate a feature vector capable of differentiating flames or distortions without relying on conventional texture descriptors. However, challenges such as poor picture/video quality or adverse weather conditions necessitate implementing modern supplementary methods to enhance current approaches.

Handcrafted feature extraction, as described above, can become complex and labor-intensive. Learning-based methods that automatically extract relevant details from images, mainly based on deep learning architectures, have consistently demonstrated improved accuracy performances and lower false alarm rates than conventional approaches. Ba et al. [30] introduced Smoke Net, a novel CNN model incorporating spatial and flow attention mechanisms for improved feature representations in visual classifications. Luo et al. [31] proposed a CNN-based approach for flame identification, leveraging the kinetic characteristics of smoke. They employed dynamic frame references from the background and foreground and a CNN architecture to extract candidate pixel highlights automatically. Zhang et al. [32] used a Faster R-CNN approach to detect wildland forest fires while additionally developing a novel technique for generating synthetic smoke images. Chaoxia et al. [33] introduced an enhanced Faster R-CNN method for flame detection by incorporating a color-guided anchoring strategy and embedded global information-guided flame detection.

Recent novel approaches target reducing the total number of model parameters by employing innovative pruning techniques. As such, the Distillation for Knowledge Transfer to Pruned Model (DK-TPM) [34] uses feature distillation with smoothing (FDS) to transfer flame features from the teacher model into the student model.

The Dynamic Deformable Adaptive Framework (DDAF) [35] dynamically detects the flame region using the so-called Deformable Convolution Network v2 to cope with deformations and scale variations of the flames.

A distinct line of research has considered transformed-based architectures and associated attention mechanisms to implement both detection and combined segmentation+classification approaches. Proposed solutions include the Swin Transformer [17], TransFire, and TransUNet architectures [36], although the computational cost is significantly higher than existing CNN variants.

You Only Look Once (YOLO) models stand as state-of-the-art object detectors due to their demonstrated speed, precision, and adaptability [10]. Built around CNNs, the first versions of the model operate by dividing an image into a grid of cells, each representing a distinct area of the image. Within this grid, a set of bounding boxes is utilized to detect objects, while a predefined set of classes is associated with these regions. Upon receiving an input image, YOLO analyzes the grid cells, categorizes them based on the objects detected within, and constructs bounding boxes around these objects, assigning them a corresponding class. Based on the PyTorch framework, the YOLOv5 model improves feature extraction by using CSPDarknet53 as the backbone and additionally offering multi-scale feature fusion capabilities. The YOLOv7 foundation layer is represented by the Extended Efficient Layer Aggregation Network (E-ELAN), which enables faster and more precise detection performances. YOLOv8 introduces additional enhancements such as anchor-free detection, larger multi-scale feature maps, and Distribution Focal Loss. YOLOv9 builds on the solid foundation of previous models by introducing two extra features, namely Programmable Gradient Information (PGI) and a Generalized Efficient Layer Aggregation Network (GELAN) [37]. YOLOv10, the most recent member of the YOLO family, introduces the innovative idea of Non-Maximum-Suppression (NMS)-free training by implementing a consistent dual assignments training method to increase speed [38]. Finally, the YOLO-NAS model [39] is based on a Neural Architecture Search (NAS) procedure that looks for optimal architectural choices regarding the number of layers, layer types, kernel sizes, and connectivity patterns.

Each YOLO model offers various tradeoffs between accuracy and speed and comes with different sizes such as nano (-n), small (-s), medium (-m), balanced (-b), large (-l), and extra-large (-x). 

Within the fire/smoke detection framework, Park et al. proposed a fire detection method for urban areas utilizing static Elastic-YOLOv3 during night-time [40]. Mukhiddinov et al. [41] proposed an early wildfire smoke detection system utilizing enhanced YOLOv5 photographs captured by unmanned aerial vehicles (UAVs). Dou et al. [42] introduced the Convolutional Block Attention Module (CBAM), BiFPN, and transposed convolution into YOLOv5, significantly enhancing the algorithm’s accuracy in fire detection. Subsequently, Du et al. [43] integrated ConvNeXt into YOLOv7, creating a fire detection model that effectively balances the detection speed and accuracy. Chen et al. [44] employed Ghost Shuffle Convolution to construct lightweight modules, reducing the model’s parameter count and accelerating the convergence speed in YOLOv7. Talaat et al. [45] proposed an enhanced method for smart city fire detection based on the YOLOv8 algorithm, capitalizing on deep learning to detect specific fire features in real time. Zhang et al. [46] proposed a change in the YOLOv8 architecture, where GhostnetV2-C2F is integrated into the algorithm’s backbone to facilitate long-range attention with minimal computational resources. The Detectron2 model has also been used for (forest) fire detection [47], showing robust performances even for small fires situated at long distances for both daylight and night-time scenarios.

## 3. Protocol for Dataset Generation

**Figure 1:**
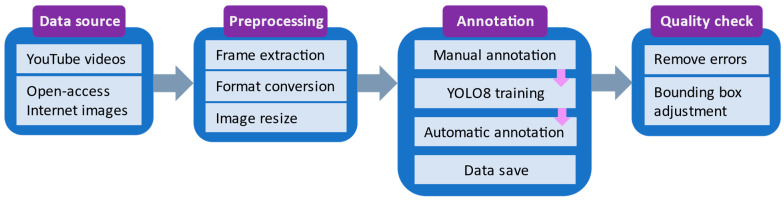
Overview of the dataset generation protocol.

Figure 1 illustrates the protocol for generating the new FireAndSmoke dataset. It includes the image collection and composition, the preprocessing steps, and the annotation procedure. Finally, the efficiency of the YOLO-type object detector family is evaluated on the new set using classical performance indicators. 

### 3.1. Dataset Composition

We collected a wide range of images from various real-world scenes, including buildings on fire, cars on fire, vegetation fires, wildfires, trash fires, and interior fires. The scenes include aerial views from drones, close-up and distant shots, as well as images captured from below, where the fire is at a higher altitude. The fire instances vary in magnitude and size and may be obscured by vegetation or other objects. The images were captured at different times throughout the day, from early morning to late night. Moreover, the images were taken under various weather conditions, including sunny, cloudy, rainy, and foggy scenarios. Overall, the comprehensive nature of the FireAndSmoke dataset, with its wide range of scenes, perspectives, sizes, and environment scenarios, makes it highly suitable for training models that can accurately and reliably detect and analyze fires and smoke presence.

The dataset includes images from over 1200 videos (manually downloaded from YouTube) and static images manually downloaded from Google, totaling 22,970 images classified into three distinct classes: fire, smoke, and other. Including the “other” class serves the purpose of labeling objects that may resemble fire or smoke but are not related to fire incidents. Instances marked as “other” include natural and artificial light sources such as sunsets/sunrises, different types of clouds, streetlamps, bonfire glows, reflections, vehicle headlights, emergency lights of various vehicles, or lights from public electronic screens, all of which could be mistaken for fire. This approach enhances the model’s ability to distinguish between relevant and irrelevant features in images, ultimately improving the overall accuracy and reliability of the fire detection system.

Details on the actual dataset composition are given below:

(a) Daylight scenes (15,812 images):
  - 2121 smoke-only scenes (thin or dense, small/medium/large size, far/medium/close distance)
  - 2475 fire-only scenes (small/medium/large size, far/medium/close distance, car/building/trash/wood burning)
  - 645 other-only scenes (different types of clouds, sunrises/sunsets, car headlights, or other types of shiny surfaces)
  - 4312 fire-and-smoke scenes (small/medium/large size, far/medium/close distance, car/building/trash/wood burning)
  - 3138 drone-captured scenes containing fire-and-smoke or just smoke-only images (small/medium/large size, far/medium/close distance, car/building/trash/wood burning)
  - 3121 vegetation fires (forest fires, grass fires, wildfires)
(b) Night-time scenes (7158 images):
  - 2657 fire-and-smoke scenes (small/medium/large size, far/medium/close distance, car/building/wood burning)
  - 1852 fire, smoke, and other scenes (small/medium/large size, far/medium/close distance, car/building burning, car lights, streetlamps, illuminated advertisement panels)
  - 1228 fire-only scenes (small/medium/large size, far/medium/close distance, car/building burning)
  - 1421 other-only scenes (small/medium/large size, far/medium/close distance, car lights, streetlamps, illuminated advertisement panels)

**Figure 2:**
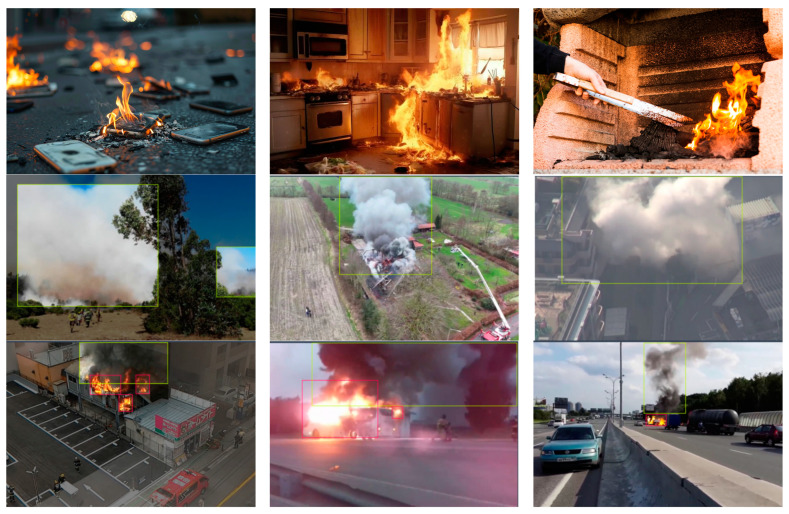
Examples of images containing fire, smoke, and combined instances in daylight images.

**Figure 3:**
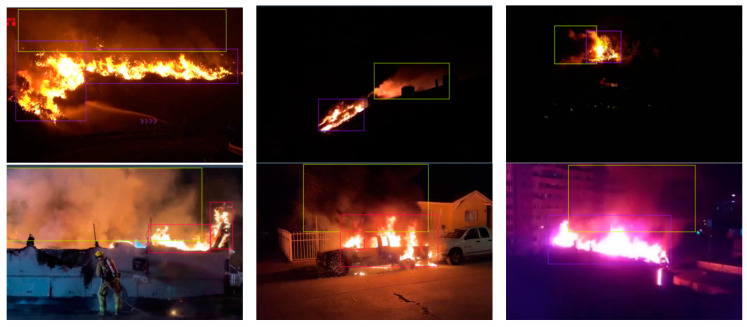
Examples of images containing fire, smoke, and combined instances in night-time images.

**Figure 4:**
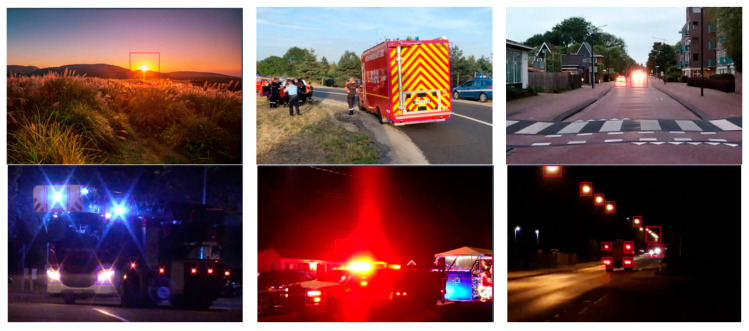
Examples of images containing “other” class instances.

Images were normalized to the dimensions required by the models, and histogram equalization was employed to compensate for the illumination variability. Augmentation techniques were applied based on horizontal flip and rotation with ±20°. Examples demonstrating the diversity of the actual images are given in Figure 2, Figure 3 and Figure 4. 

### 3.2. Data Labeling Procedure

To avoid manually annotating all collected images, we adopted a multi-step approach. Initially, we annotated 1000 images containing fire, smoke, and other instances, creating a small dataset hosted on the Roboflow platform [48]. We then trained a YOLOv8m model using this dataset to identify fire and smoke instances. Subsequently, we used this trained model to run inferences on the remaining images. The bounding box coordinates and labels generated by the model during this prediction process were saved as. txt files (using the YOLOv8 format). The images in the dataset and the corresponding labels were uploaded on the Roboflow platform, where we reviewed all the annotated images to ensure the accuracy of bounding boxes and labels. Any incorrectly annotated images were removed, and adjustments were made to bounding box dimensions or locations as needed. Additionally, any labeling errors were corrected to guarantee the robustness, quality, and reliability of the dataset.

We built on the availability of a broad range of tools that Roboflow provides for data conversion and preprocessing, further streamlining the training process. The images were resized to a consistent size of 640 × 640 pixels to ensure uniformity across the dataset. Once the dataset was successfully reviewed, we exported it in various formats, such as YOLOv5–v8 PyTorch. The same dataset format as YOLOv8 can be used for YOLO-NAS training, ensuring compatibility across different YOLO models.

## 4. Fire/Smoke Detection Evaluation

Detection performances were assessed in terms of speed (expressed as the number of frames per second), class-wise Precision (*P*), Recall (*R*), and Mean Average Precision (mAP@50) evaluated at both 50% and in the 50–95% IoU (intersection-over-union) range for the three classes under study. The IoU value (also known as the Jaccard index) quantifies the degree of overlap between two bounding boxes (e.g., the ground truth and the one generated by an object detection algorithm) as the ratio between their corresponding intersection and reunion areas, respectively.

Precision and Recall parameters are defined as follows:

(1) $$P\;=\;\frac{TP}{TP\;+\;FP};\;R\;=\;\frac{TP}{TP\;+\;FN}$$

where *TN* is the true negative rate, *FN* is the false negative rate, *TP* is the true positive rate, and *FP* is the false positive rate.

When the overlap between the predicted box and the ground truth bounding box exceeds a given threshold value (e.g., IoU > 50%), the detection is considered correct, otherwise it is counted as a false positive. If the predicted box around an actual fire/smoke instance is missing, we get a false negative case. The F1-score, defined as the harmonic mean of Precision and Recall, may offer valuable information in cases exhibiting a significant imbalance between the number of examples available in the categories under study.

The Precision–Recall curve (computed for a given IoU threshold value) illustrates the relationship between these two metrics for different confidence scores associated with the instance detections of a specific object provided by a given model, while the area under the curve (denoted as AP) quantifies the performance of the model on this task. The Mean Average Precision (mAP) calculates the average precision across multiple classes or categories and provides a single scalar value that summarizes the overall model performance. 

All experiments were conducted on an RTX 4090/24GB GPU running PyTorch models. Details on the experimental setup parameters are given in Table 2 and Table 3. The data split is indicated in Table 4. 

Table 5 presents comparative performance results on the test set images for the seven models considered, showing a mild superiority of YOLOv8 and YOLOv10 variants. mAP@50 values are situated in the range exhibited by other models evaluated on different datasets [7] and speed performances indicate real-time operation.

**Figure 5:**
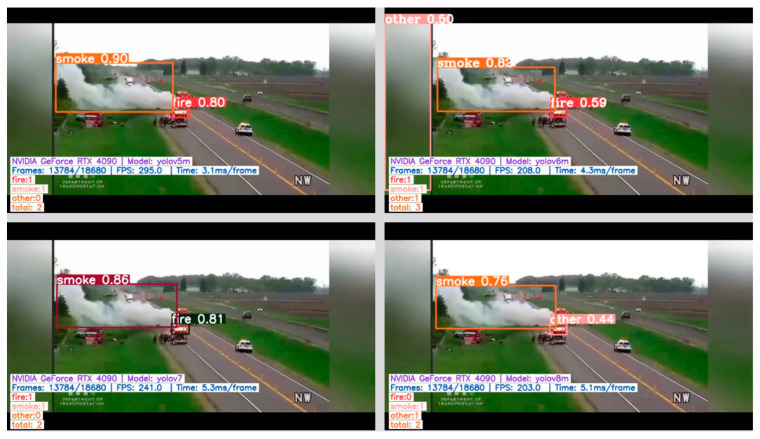

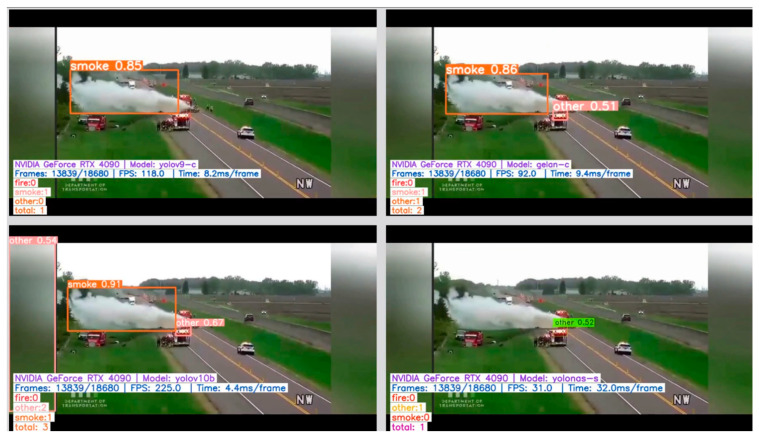
Examples of correct “smoke” detection by YOLO-type models and false positive “fire” detection by YOLOv5–v7 models.

**Figure 6:**
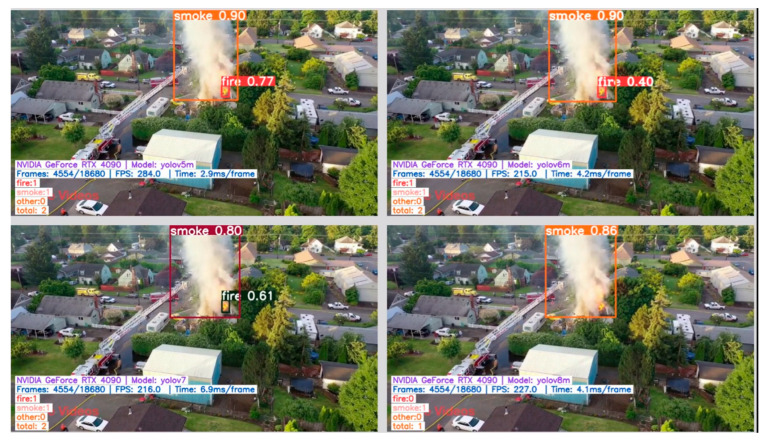

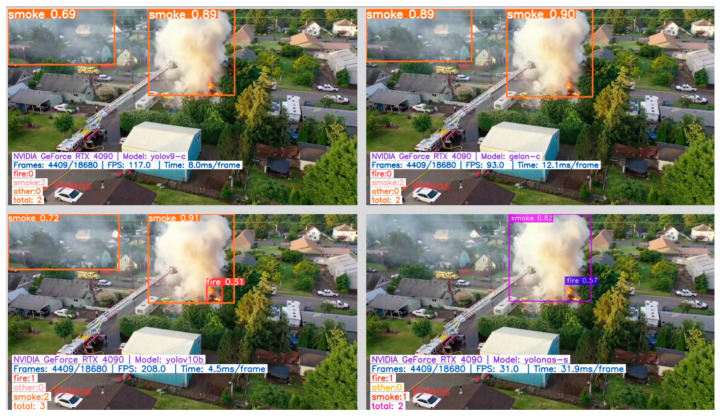
Examples of correct “fire” and “smoke” detections by YOLO-type models, except for YOLOv8 which misses the fire instance detection.

**Figure 7:**
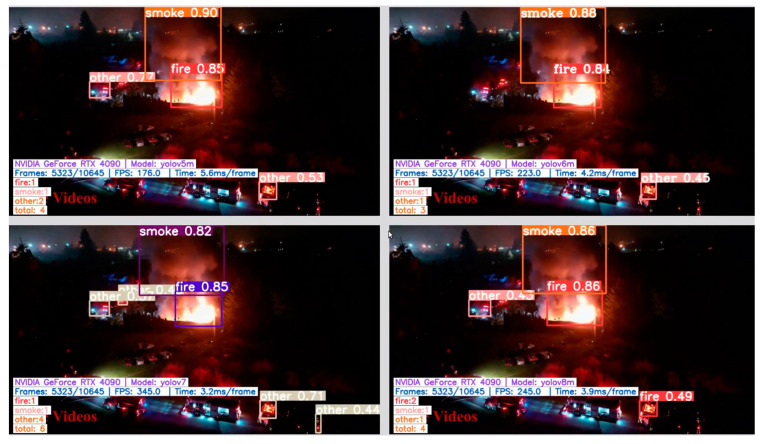

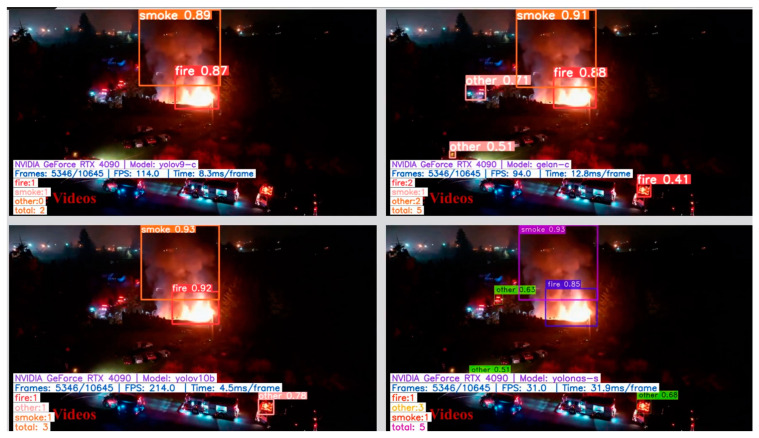
Examples of correct “fire”, “smoke”, and “other” instances detection by the YOLO-type models, except for YOLOv8 which generates a false positive “fire” detection.

**Figure 8:**
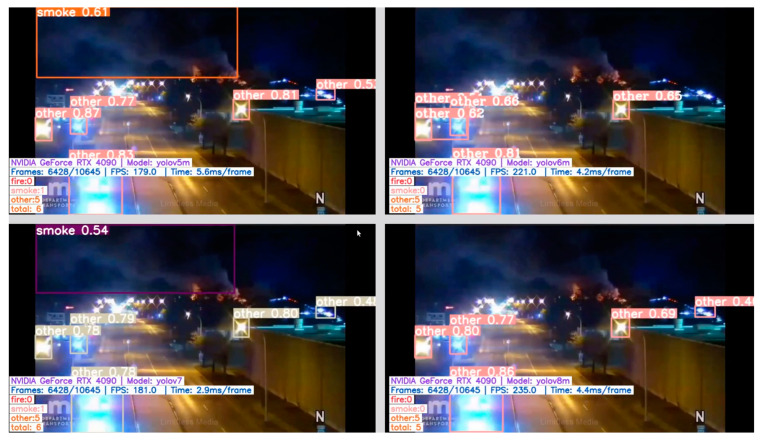

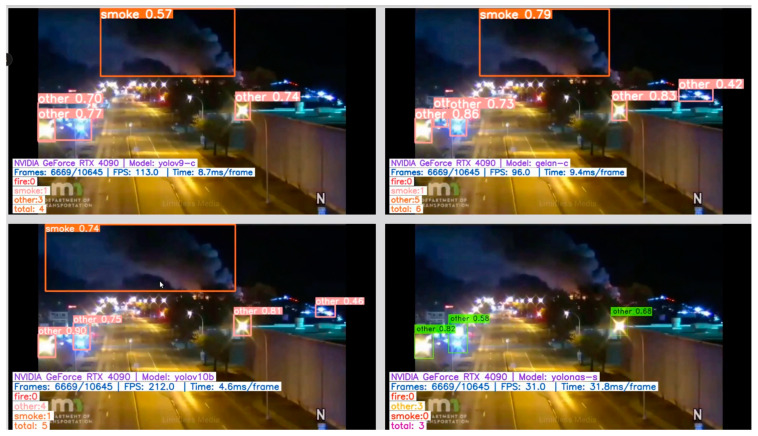
Challenging night-time scenes showing correct “smoke” and “other” class detection by YOLO-type models, while YOLO-NAS misses detecting the smoke instance.

Examples of correct fire/smoke detections are given in Figure 5, Figure 6, Figure 7 and Figure 8. The figures illustrate the best performances and false detections made by the models under study.

To better understand the comparative performances of the models under study, we generated two test video sequences containing challenging scenarios that are difficult to evaluate even by human subjects. Those are called *VideoDayMixt.mp4* (18,680 frames) and *VideoNightMixt.mp4* (10,645 frames) and are hosted on the GitHub page of the project [9]. We additionally provide scripts implementing detailed performance metrics measuring the total number of detections per class, the average confidence score of the detections per class within specific value ranges (e.g., the number of detections for a specific class with a confidence score above 0.8), and the total number of incorrect predictions made by the models. The performances are reported in Table 6 and Table 7. 

## 5. Discussion

It is difficult to appropriately compare the performances of different models introduced in the literature since they are not typically evaluated on the same dataset, whose composition may vary significantly in terms of dimensionality, diversity, resolution, or background complexity. Nevertheless, the experimental results indicated previously revealed that all models achieved high detection performances, with mAP@50 values reaching 91% for the “fire” and “smoke” classes. As shown in Table 8, those compare favorably with existing results, although some papers only consider fire or smoke detection separately and seldom use “other” class instances.

All the evaluated models successfully coped with realistic situations where other light sources resembling fire may translate into false positive predictions. Moreover, all models exhibited real-time operation, with inference speeds above 100 fps. Overall, the YOLOv5 model performed fastest on inference tasks, while the YOLOv10 model provided the most robust results, reliably detecting fire and smoke instances in images affected by significant variations in scale and illumination conditions. It performed best even for very difficult smoke detection situations, where the background or the sky raises serious challenges. Despite being slower than the other variants, YOLO-NAS excelled in predicting fire instances at high distances, confirming its known capacity for identifying small objects.

Regarding the training time, YOLOv7 required the longest duration (≈8 h) due to its high number of parameters (about 36 million). YOLO-NAS trained for about 6 h, although its number of parameters is much smaller (19 million). This may be attributed to the expansive search space that covers various architectural configurations and hyperparameter settings.

The performance evaluation of the models on the challenging daylight and night-time videos revealed that YOLOv5 and YOLOv10 offered around 0.8 precision scores. Moreover, both models exhibited high-confidence detection rates, making them ideal for critical applications such as monitoring residential areas or industrial settings where timely and accurate detection can prevent disasters. The results suggest that YOLOv10 yields the highest precision on detected scenes, a much-desired trait for cases when false positives must be minimized, such as deploying first responders at distant or dangerous locations.

In terms of detection volume, YOLOv6 and YOLOv7 were notable. On the two test videos, YOLOv6 recorded the highest number of detections, with 14,635 for fire and 22,145 for smoke, albeit at lower precision scores of 0.730 and 0.766, respectively. YOLOv7 logged 14,761 fire detections and 21,895 smoke detections with slightly better precision scores of 0.770 and 0.758, respectively. These models are suited for applications prioritizing coverage over accuracy, such as vast industrial complexes or forest areas where missing a single event could have severe consequences.

YOLOv6 and YOLOv8 provided a balanced approach with a good number of detections and acceptable precision levels. These models are suitable for general surveillance and monitoring, where a balance between detection volume and accuracy is needed.

Explainable artificial intelligence (XAI) may increase the transparency and reveal the decision-making operation of various learning-based models (including the source of incorrect behavior), thus paving the way for their broad application in real-life tasks. While the literature on this topic related to classification tasks is quite large, the references focusing on object detection are relatively scarce. 

This is mainly due to the essential differences between classification and object detection tasks. While the former uses (single) categorical output objectives, the second employs regression-type targets that consider the object class probabilities, the localization information, and the objectness score, directly affecting defining proper similarity metrics that account for all those components. 

Significant XAI approaches for object detection include model agnostic solutions such as Detector-Randomized Input Sampling for Explanation of Black-box Models (D-RISE) [49] and the Gaussian Class Activation Mapping Explainer (G-CAME) [50].

Identified as a random masking perturbation-based approach, the D-RISE model builds on the success of the previously introduced RISE [51] technique to yield saliency maps that would explain detection decisions. The method uses masks to separate particular regions of an input image, passes the masked images through the analyzed model, and assesses the importance of each area based on a weighting score associated with each mask that combines both the object class probabilities and the localization information. 

G-CAME is a Class Activation Mapping (CAM)-based method that also yields a saliency map for each detected object. The algorithm operates by adding a Gaussian kernel as the weight for each pixel in the activation maps of specific layers in the model’s architecture and is more performant and computationally faster than D-RISE.

**Table 8:** Performance comparison with other fire/smoke detection systems.

System	Model	Precision	Recall	F1	mAP@50	Dataset Size
Tian [52]	YOLOv5n	0.92	0.91	0.91	0.94	>10,000
Avazov [53]	YOLOv7	0.97	0.94	0.96	0.97	4622
Talaat [45]	YOLOv8	0.93	0.94	0.94	0.97	>25,000
Luo [31]	YOLOX	0.86	0.72	0.78	0.84	5000
Geng [54]	YOLOFM	0.94	0.94	0.94	0.97	18,644
This work	YOLO10	0.86	0.85	0.85	0.91	>22,000

## 6. Conclusions

The paper introduces a new annotated image dataset including various fire and smoke scenarios derived from real-world events. These encompass diverse settings such as indoor and outdoor fires and both small and large-scale flames/smoke, under largely varying lighting conditions. In order to minimize false positive detections, the dataset incorporates “other class” examples of non-fire or non-smoke scenarios, forcing the models to accurately discriminate between genuine and false instances of fire and smoke.

The comparative performance results of the YOLO family of object detectors reported in the previous sections revealed that each model might find utility for specific operational requirements, ensuring optimal performance in diverse surveillance and safety environments. The analysis concluded that YOLOv5 and YOLO10 are most suitable for applications requiring high accuracy and reliability due to their superior precision and confidence detection rates in both fire and smoke scenarios. While YOLOv10 is recommended when minimizing false positives is paramount, YOLOv7 offers advantages in scenarios requiring many detections.

Integrating deep learning models within a video processing pipeline may facilitate the automatic analysis of surveillance footage, enabling timely response and mitigation actions. We have performed preliminary tests with an end-to-end solution that integrates our YOLO-based fire detection models within the DeepStream framework from Nvidia [55]. Images captured by (visible spectrum) cameras are transmitted through a 5G communication network to a processing server that yields a dashboard that includes valuable insights for the authorized users (firefighters, emergency services). The application will report fire incidents, the actual location of the incident, and the real-time video flow from the location.

Additional comparative experiments will be conducted considering transformer-based alternatives and ensemble learning methods. To effectively use examples of images that may resemble (but not actually represent) fire instances, we will consider a contrastive learning approach (e.g., by means of Siamese-type networks) that has proved effective in classification and object tracking tasks. One essential subject to be addressed is a comparative analysis of the effect of explainability methods on the models under study. From a practical perspective, an exciting research topic could consider the efficiency of the various fire and smoke detection approaches in adverse weather conditions (rain, fog, and snow).

## Figures and Tables

**Table 1:** Fire/smoke open datasets.

Dataset	Classes	Num Images	Source	Content
Foggia et al. [11]	Fire/Non-fire	62,690	Ground cameras’ video frames	Indoor/outdoor scenes
Li et al. [12]	Fire/Non-fire	50,000	Ground cameras’ video frames and static images	Indoor/outdoor scenes
Yar et al. [13]	Fire/Non-fire	3804	Ground cameras’ video frames	Outdoor scenes
Dilshad et al. [14]	Fire/Non-fire	47,124	Ground cameras’ images	Foggy, low-light outdoor scenes
Shamsoshoara et al. [15]	Fire/Smoke/Other	39,375	Air-borne visible spectrum/infrared cameras	Forest fires
Yar et al. [16]	Fire/Non-fire	6000	Air/space-borne cameras	Haze, fog, night outdoor scenes
Wang et al. [17]	Fire/Smoke/Other	122,634	Ground/air-borne cameras	Indoor/outdoor scenes

**Table 2:** Experimental setup parameters.

Parameter Name	Type
Operating system	Ubuntu 22.04.3 LTS
CPU	Intel Core 9 Gen i9-13900KF (5.8 GHz)
GPU	RTX 4090 24 GB
RAM	DDR5 64 GB (6000 MHz)
Deployment environment	Python 3.10.2
Deep learning framework	PyTorch 2.1.2
Accelerated computing architecture	CUDA 12.1

**Table 3:** Training parameters of the deep learning models.

	YOLOv5	YOLOv6	YOLOv7	YOLOv8	YOLOv9	YOLOv10	YOLO-NAS
Epochs	100	100	100	100	100	100	100
Batch size	64	44	32	64	16	32	64
Optim. alg.	SGD	SGD	SGD	SGD	SGD	SGD	Adam
Model weights	yolov5m	yolov6m	yolov7	yolov8m	yolov9-c	yolov10m	yolo_nas_s
Num. params. (million)	21.2	34.9	35.9	25.9	50.7	19.1	19

**Table 4:** The train/validation/test dataset split for the experiments.

	Train	Validation	Test
Nr of images	20,108	4757	2417
Fire instances	19,864	4490	2353
Smoke instances	18,017	4282	2249
Other instances	10,648	2691	1332

**Table 5:** The detection performances of the deep learning models on the test set.

Model	Fire					Smoke					Other					Speed (fps)
	P	R	mAP @50	mAP @50–95	F1	P	R	mAP @50	mAP @50–95	F1	P	R	mAP @50	mAP @50–95	F1	
YOLOv5	0.86	0.86	0.91	0.69	0.86	0.88	0.82	0.89	0.66	0.85	0.80	0.78	0.84	0.69	0.79	210
YOLOv6	0.84	0.86	0.91	0.64	0.85	0.89	0.81	0.91	0.63	0.84	0.78	0.73	0.81	0.56	0.75	215
YOLOv7	0.83	0.88	0.91	0.64	0.85	0.87	0.84	0.90	0.62	0.86	0.76	0.79	0.84	0.58	0.77	230
YOLOv8	0.86	0.85	0.91	0.69	0.85	0.88	0.84	0.91	0.68	0.85	0.82	0.77	0.85	0.63	0.79	230
YOLOv9	0.85	0.85	0.91	0.69	0.85	0.88	0.85	0.91	0.68	0.85	0.76	0.68	0.84	0.63	0.72	115
YOLOv10	0.86	0.85	0.91	0.69	0.85	0.89	0.83	0.91	0.68	0.86	0.82	0.74	0.84	0.63	0.78	210
YOLO-NAS	-	-	0.84	-	-	-	-	0.80	-	-	-	-	0.70	-	-	-

**Table 6:** Comparative performances of deep learning models on *VideoDayMixt.mp4* video.

		YOLOv5	YOLOv6	YOLOv7	YOLOv8	YOLOv9	YOLOv10	YOLO-NAS
		Confidence score > 0.5						
Number of detections (Average confidence score)	Fire	13,025 (0.799)	14,635 (0.730)	14,761 (0.770)	12,703 (0.743)	12,874 (0.719)	12,968 (0.769)	15,414 (0.717)
	Smoke	21,694 (0.792)	22,145 (0.766)	21,895 (0.758)	20,948 (0.768)	20,204 (0.759)	20,733 (0.796)	17,439 (0.722)
		Confidence score > 0.8						
Number of detections (Average confidence score)	Fire	8937 (0.882)	5570 (0.885)	7949 (0.883)	4762 (0.865)	2733 (0.876)	6715 (0.885)	4447 (0.891)
	Smoke	13,033 (0.892)	11,530 (0.890)	10,303 (0.885)	11,954 (0.880)	10,227 (0.887)	13,389 (0.895)	5405 (0.885)
Speed (fps)		207	214	179	197	116	210	31

**Table 7:** Comparative performances of deep learning models on *VideoNightMixt.mp4* video.

		YOLOv5	YOLOv6	YOLOv7	YOLOv8	YOLOv9	YOLOv10	YOLO-NAS
		Confidence score > 0.5						
Number of detections (Average confidence score)	Fire	7053 (0.836)	7472 (0.772)	7533 (0.796)	7114 (0.786)	6358 (0.778)	6606 (0.835)	5408 (0.781)
	Smoke	6105 (0.744)	5777 (0.673)	6152 (0.693)	4752 (0.722)	5181 (0.721)	4621 (0.743)	3020 (0.720)
		Confidence score > 0.8						
Number of detections (Average confidence score)	Fire	5540 (0.891)	4216 (0.889)	4981 (0.889)	4762 (0.865)	3716 (0.883)	5086 (0.896)	2968 (0.890)
	Smoke	2699 (0.889)	1705 (0.887)	1867 (0.881)	1959 (0.880)	2107 (0.890)	2214 (0.893)	940 (0.892)
Speed (fps)		180	219	245	240	114	216	31

## Data Availability

Data and accompanying Python code for downloading are freely available on the GitHub platform: https://github.com/CostiCatargiu/NEWFireSmokeDataset_YoloModels (accessed on 26 July 2024).
